# Understanding the genetic basis of resistance to maydis leaf blight and maturity-related traits in corn

**DOI:** 10.3389/fpls.2025.1551940

**Published:** 2025-03-26

**Authors:** Bhupender Kumar, Pushpendra, Santosh Kumar, Pardeep Kumar, Rakhi Singh, Shrikant Yankanchi, Debjyoti Sarkar, Chhavi Nath, Bahadur Singh Jat, Pravin Kumar Bagaria, Sumit Kumar Aggarwal, Prahlad Piyal, S. B. Singh, Sujay Rakshit, H. S. Jat

**Affiliations:** ^1^ Indian Council of Agricultural Research (ICAR)-Indian Institute of Maize Research, Ludhiana, Punjab, India; ^2^ Indian Council of Agricultural Research (ICAR)-Indian Agricultural Research Institute, Hazaribag, Jharkhand, India; ^3^ Indian Council of Agricultural Research (ICAR)-Indian Institute of Agricultural Biotechnology, Ranchi, Jharkhand, India

**Keywords:** generation mean analysis, heritability, resistance, susceptible, correlation, disease

## Abstract

Maize is one of the most versatile and commercially produced crops used for food, feed, fodder, ethanol, oil, and industrial raw materials. Maize is affected by various diseases, but among these, maydis leaf blight (MLB) is one of the most serious diseases. The disease is caused by *Cochliobolus heterostrophus* and is responsible for yield losses up to 40%. When developing cultivars for a specific ecology, days to flowering and maturity are important breeding traits to consider. Thus, understanding the genetic basis of MLB resistance, specifically the “O” race of the pathogen, and maturity-related traits is crucial to develop climate-resilient maize hybrids. This study aimed to determine the gene actions and their interactions for MLB resistance and maturity-related traits using a six-parameter model (P_1_, P_2_, F_1_, BC_1_P_1_, BC_1_P_2_, and F_2_). Five experimental crosses were attended using resistant (R) (*CML269-1* and *P72c1Xbrasil1177-2*) and susceptible (S) (*HKIPC4B* and *ESM113*) lines in R×S (1), S×R (2), R×R (1), and S×S (1) combinations. The susceptible lines belonged to the early (*HKIPC4B*) and medium (*ESM113*) maturity groups, while the resistant lines belonged to the medium (*CML269-1*) and late (*P72c1Xbrasil1177-2*) maturity groups. These six genetic populations were screened under artificially created epiphytotic conditions at a hot-spot site. In the analysis, MLB resistance showed a dominance genetic effect with significant (*P*<0.01) additive × additive interactions. Maturity-related traits showed significant dominance genetic effects (P< 0.01), with dominance × dominance interactions, suggesting the suitability of hybrid breeding for these traits. The estimated genes responsible for MLB resistance ranged from 0.002 to 5.78 per cross. In MLB resistance, broad and narrow-sense heritability were found to be 91.9% and 84.3%, respectively, which indicated the possibility of genetic improvement through selection. Disease response and maturity-related traits were negatively correlated, suggesting that long-duration genotypes are more resistant to disease than short-duration. The detailed understating of gene actions can aid in designing breeding strategies to develop resistant cultivars with the required duration for various stress-prone ecologies.

## 1 Introduction

Following rice and wheat, maize is the third most important cereal crop. It has a wide range of uses, including food, feed, fodder, ethanol, and oil. The top maize-growing countries in the world include the United States, China, Brazil, Argentina, Ukraine, and India. In India, maize is cultivated on 10.7 million hectares, with a total production and average productivity of 38 million tons and 3.5 tons/ha, respectively (FAOSTAT, 2023). The extensive biotic and abiotic factors lead to significant yield losses in maize during both its growth and reproductive phases. Among the biotic stresses, maydis leaf bight is a severe fungal leaf disease affecting maize and other crops, such as sorghum and teosinte, globally. Severe attacks of MLB can reduce crop yields by up to 40% (Malik et al., 2018; Aggarwal et al., 2024). In India, MLB has emerged as one of the most widespread and severe diseases, particularly affecting regions such as Jammu and Kashmir, Himachal Pradesh, Sikkim, Meghalaya, Punjab, Haryana, Rajasthan, Delhi, Uttar Pradesh, Bihar, Madhya Pradesh, Gujrat, Maharashtra, Andhra Pradesh, Karnataka, and Tamil Nadu. This disease thrives under warm temperatures (20°C–32°C) with moderate to high humidity (>80%), posing a significant threat to maize production globally and in India specifically (Jeevan et al., 2020).

The pathogen *Cochliobolus heterostrophus* consists of three physiological races: O (predominant race), T (Texas), and C (Charrua). Race T, according to the type of sterile cytoplasm, is prevalent in the USA and caused a major epidemic in 1970 due to the widespread use of Cytoplasmic Male Sterile (CMS)-T cytoplasm-based hybrids (Ullstrup, 1972). The races O and T produce the Hm-O and Hm-T phytotoxins, respectively, and create varying degrees of virulence (Nisa et al., 2024). Race C, primarily found in China, infects maize with the CMS-C cytoplasm (Wei et al., 1988). Race O is widespread in the USA, India, Africa, and Western Europe, infecting all susceptible maize cultivars regardless of cytoplasm type (Balint-Kurti et al., 2007). These races illustrate regional prevalence and cytoplasm-specific pathogenicity in maize. Protective fungicides are ineffective under high MLB disease pressure, especially in regions with susceptible maize cultivars (Hooda et al., 2018). Genetic host resistance offers a more sustainable approach, though research on resistance genes for race O is limited. No maize cultivars are completely immune to MLB, but some inbred lines show significant resistance (Sharma and Rai, 2005; Kumar et al., 2016; Manjunatha et al., 2019). The lack of immune lines or complete resistance genes has pushed breeders to focus on polygenic and quantitative resistance breeding. This strategy remains the most viable option for managing MLB effectively.

Maize possesses considerable potential for harboring disease-resistant genes. Identifying sources of resistance and accumulating these genes are critical steps for selecting superior genotypes in breeding programs. Hence, comprehensive research should be conducted to combat this disease in all significant maize-producing regions. MLB resistance is predominantly quantitative, which has important implications for developing resistant maize varieties (Kumar et al., 2016). Several studies have suggested that maize has an overdominance type of gene action in days to tasseling and a partial dominance type in days to silking (Sher et al., 2012). However, limited reports are available on study of gene action in Indian-adapted tropical maize germplasm.

Generation mean analysis represents a valuable method for estimating the primary genetic effects (additive, dominance) and their interactions (digenic interactions) that influence quantitative traits, including disease resistance (Shashikumar et al., 2010: Akbar et al., 2018). This approach provides insights into the genetic effects, such as additive, dominance, and epistasis [additive × additive (i), additive × dominance (j), and dominance × dominance (l)], that govern the inheritance of traits. These genetic effect estimates are crucial for devising effective breeding strategies for segregating generations. Thus far, most of these studies have been conducted in temperate genetic backgrounds, however, a few studies have been carried out on these targeted traits in tropical maize, primarily in India (Kumar et al., 2016). Developing MLB-resistant maize cultivars with the required flowering and maturity days, and understanding the nature and extent of their genetic action is essential for systematic breeding (Jeevan et al., 2020). Thus, tropical-adapted maize germplasm has been used to study the genetic effects and nature of gene action for MLB resistance, days to flowering, and maturity. The objective of this study was to determine the gene actions for the MLB resistance and maturity-related traits using a six-parameter (P1, P2, F1, BC1P1, BC1P2, and F2) model of generation mean analysis. Furthermore, we aimed to establish the connection between MLB resistance and crop duration/maturity.

## 2 Materials and methods

### 2.1 Development of plant genetic materials

The current investigation was initiated in 2020 with the selection of contrasting parents, including resistant (R), i.e., CML269-1 (medium duration), and P72c1Xbrasil1177-2 (long duration) and susceptible (S), i.e., HKIPC4B (short duration) and ESM113 (medium duration), lines from previously published reports (Kumar et al., 2016) for MLB disease, flowering, and days to maturity (**Supplementary Table 1**) during Kharif 2020. The different maturity group lines were planted in a staggered manner to match the flowering to generate F_1_s and other populations. The plants were selected and used to generate F_1_s during Rabi 2020–2021 and then F_2_s and backcrossed populations during *Kharif* 2021. As a result, five experimental crosses were generated using the R lines (CML269-1 and P72c1Xbrasil1177-2) and S lines (HKIPC4B and ESM113) in R×S (one cross), S×R (two cross), R×R (one cross), and S×S (one cross) combinations. The susceptible lines belonged to the short (*HKIPC4B*) and medium (*ESM113*) duration groups, while the resistant lines belonged to the medium (*CML269-1*) and late (*P72c1Xbrasil1177-2*) duration groups. In the second season, all five hybrids were planted with their parents in a staggered manner and each F_1_s hybrid was selfed to generate F_2_s population. Furthermore, all F_1_ were backcrossed with both the respective parents (P_1_ and P_2_), to generate backcrosses (BC_1_P_1_ and BC_1_P_2_). In the first season, the F_1_ seeds were kept as a backup to use in the evaluation trials in the third year (Kharif season 2022) at the Delhi location (weather data attached in **Supplementary Table 3**).

### 2.2 Evaluation for MLB disease and flowering traits

During Kharif 2022, six genetic populations (P_1_, P_2_, F_1_, F_2_, BCP_1_, and BCP_2_) from each cross were planted and evaluated for disease under artificially created epiphytotic conditions at a hot-spot site (New Delhi). Four rows of 3-meter lengths for each parent, F_1_, and complete backcrosses and F_2_s population seeds of every cross were planted in the field. We maintained a row-to-row spacing of 65 cm and a plant-to-plant spacing of 20 cm. The standard package of practices was followed to raise a healthy crop (Bamboriya et al., 2020). Artificial inoculation was performed according to Carson et al. (2004). In a conical flask containing nearly 45 grams of sorghum grains, *Cochliobolus heterostrophus* race ‘O’ was cultured, which is predominantly found in Indian maize (Gogoi et al., 2014) and was isolated from a New Delhi location. The sorghum grains were soaked in water for 3–4 hours and then excess water was drained off. After autoclaving twice, it was seeded with fungus under aseptic conditions and incubated at 25°C –27°C for 15 days. For uniform growth on grains, the flasks were shaken once every 2–3 days. Approximately a fortnight after incubation, the material was dried at room temperature under shade on clean paper sheets. The grains were ground into a fine powder, which was then used for inoculation. The field was kept adequately moist by providing irrigation so as to commence the fungal growth. The first inoculation was conducted 35 days after sowing. The inoculation was repeated 10 days after the first inoculation to avoid any chance of disease escape. A minimum of 10 plants in the parent and F_1_ populations and all the F_2_s and backcross population plants were rated on a disease scale (1 = highly resistant and 9 = highly susceptible) after the grain-filling stage (Hooda et al., 2018; **Supplementary Table 2**). The disease symptoms developed after inoculation (**Supplementary Figure 1**). The percent disease incidence (PDI) was calculated for MLB as per Hooda et al. (2018) and utilized in this genetic study. In addition to disease scoring, days to 50% anthesis, silking, and physiological maturity observations were also recorded in the same trials. The days to 50% anthesis (anther dehiscence) and days to 50% silking (silk emergence) are the number of days from the sowing to 50% of the plants flowering on a plot. Similarly, physiological maturity was recorded as the number of days from sowing to 75% dry husk of cobs of all the plants on a plot.

### 2.3 Biometrical analysis

All the statistical analyses were carried out using a DOS-based program in the TNAUSTAT statistical package (Manivannan, 2014).

The scaling tests (A, B, C, and D) were determined based on the approach proposed by Mather (1949), which involves simple linear combinations as detailed below:


(1) $$\text{ScaleA}=2{\bar{\text{BCP}}}_{1}-{\bar{\text{P}}}_{1}-\bar{\text{F}}$$

(2) $$\text{Scale B}=2{\bar{\text{BCP}}}_{2}-{\bar{\text{P}}}_{2}-{\bar{\text{F}}}_{1}$$

(3) $$\text{Scale C}=4{\bar{\text{F}}}_{2}-2{\bar{\text{F}}}_{1}-{\bar{\text{P}}}_{1}-{\bar{\text{P}}}_{2}$$

(4) $$\text{Scale D}=2{\bar{\text{F}}}_{2}-{\bar{\text{BCP}}}_{1}-{\bar{\text{BCP}}}_{2}$$

Where, ${\bar{\text{P}}}_{1}$ , ${\bar{\text{P}}}_{2}$ , $\;{\bar{\text{F}}}_{1}$ , ${\bar{\text{F}}}_{2}$ , ${\bar{\text{BCP}}}_{1}$ and ${\bar{\text{BCP}}}_{2}$ are means of different generations, respectively.

The variances of the quantities A, B, C, and D were determined using the variances from different generations, as outlined below:


(5) $$\text{VA}=4\text{V }({\bar{\text{BCP}}}_{1})+\text{V }({\bar{\text{P}}}_{1})+\text{V }({\bar{\text{F}}}_{1})$$

(6) $$\text{VB}=4\text{V}({\bar{\text{BCP}}}_{2})+\text{V }({\bar{\text{P}}}_{2})+\text{V }({\bar{\text{F}}}_{1})$$

(7) $$\text{VC}=16\text{V }({\bar{\text{F}}}_{2})+4\text{V }({\bar{\text{F}}}_{1})+\text{V }({\bar{\text{P}}}_{1})+\text{V }({\bar{\text{P}}}_{2})$$

(8) $$\text{VD}=4\text{V}({\bar{\text{F}}}_{2})+V\;({\bar{BCP}}_{1})+V\;({\bar{BCP}}_{2})$$

Where, VA, VB, VC, and VD are the variances of the respective scales A, B, C and D; $\text{V}{\bar{\text{P}}}_{1}$ , $\text{V}{\bar{\text{P}}}_{2}$ , $\text{V}{\bar{\text{F}}}_{1}$ , $\text{V}{\bar{\text{F}}}_{2}$ , $\text{V}{\bar{\text{BCP}}}_{1}$ and $\text{V}{\bar{\text{BCP}}}_{2}$ are the variances of the P_1_, P_2_, F_1_, F_2_, BCP_1_, and BCP_2_ generations, respectively.

The standard errors for the A, B, C, and D scales were calculated by calculating the square root of their respective variances. The t-test was used to assess the deviation from a hypothetical value of ‘0’. The calculated t-values were compared against the critical values from the t-distribution table at the 5% and 1% significance levels, considering the appropriate degrees of freedom.

The scaling test results were significant for all four traits in all five crosses. This suggests that the additive (*d*) and dominance (*h*) effects of genes (simple additive dominance model) are inadequate to explain the inheritance patterns of these traits. Consequently, it appears to be three type non-allelic interactions *viz.*, additive × additive (*i*), additive × dominance (*j*) and dominance × dominance (*l*) for all four traits. Therefore, it was essential to include parameters that account for non-allelic gene interaction effects, as described by Hayman (1958) in the six-parameter model of generation mean analysis. The calculations for all epistatic interactions were done as per the details given below.


(9) $$m=\text{Mean}={\bar{\text{F}}}_{2}$$

(10) $$d=\text{Additive effect}={\bar{\text{BCP}}}_{1}-{\bar{\text{BCP}}}_{2}$$

(11) $$\begin{array}{c} h=\text{Dominance effect}\\ ={\bar{\text{F}}}_{1}-4{\bar{\text{F}}}_{2}-(1/2)\;{\bar{\text{P}}}_{1}-(1/2)\;{\bar{\text{P}}}_{2}+2{\bar{\text{BCP}}}_{1}+2{\bar{\text{BCP}}}_{2} \end{array}$$

(12) $$i=\text{Additive}\times \text{Additive effect}=2{\bar{\text{BCP}}}_{1}+2{\bar{\text{BCP}}}_{2}-4{\bar{\text{F}}}_{2}$$

(13) $$\begin{array}{c} j=\text{Additive}\times \text{Dominance effect}\\ ={\bar{\text{BCP}}}_{1}-(1/2)\;{\bar{\text{P}}}_{1}-{\bar{\text{BCP}}}_{2}+(1/2)\;{\bar{\text{P}}}_{2} \end{array}$$

(14) $$\begin{array}{c} l=\text{Dominance}\times \text{Dominance effect}\\ ={\bar{\text{P}}}_{1}-{\bar{\text{P}}}_{2}+2{\bar{\text{F}}}_{1}+4{\bar{\text{F}}}_{2}-4{\bar{\text{BCP}}}_{1}-4{\bar{\text{BCP}}}_{2} \end{array}$$

Where, ${\bar{\text{P}}}_{1}$ , ${\bar{\text{P}}}_{2}$ , $\;{\bar{\text{F}}}_{1}$ , ${\bar{\text{F}}}_{2}$ , ${\bar{\text{BCP}}}_{1}$ and ${\bar{\text{BCP}}}_{2}$ are the means of different generations, respectively. Further, the variance of gene effects was calculated using the following formulas:


(15) $$\text{V}m=\text{V}({\bar{\text{F}}}_{2})$$

(16) $$\text{V}d=\text{V }({\bar{\text{BCP}}}_{1})+V\;({\bar{BCP}}_{2})$$

(17) $$\begin{array}{c} \text{V}h=\text{V }\left({\text{F}}_{1}\right)+16\text{V }({\bar{\text{F}}}_{2})+(1/4)\text{V }({\bar{\text{P}}}_{1})+(1/4)\text{ V }({\bar{\text{P}}}_{2})\\ +4\text{V }({\bar{\text{BCP}}}_{1})+4\text{V }({\bar{\text{BCP}}}_{2}) \end{array}$$

(18) $$\text{V}i=4\text{V }({\bar{\text{BCP}}}_{1})+4V\;({\bar{\text{BCP}}}_{2})+16V\;({\bar{F}}_{2})$$

(19) $$\text{V}j=\text{V }({\bar{\text{BCP}}}_{1})+(1/4)\;V\;({\bar{P}}_{1})+\text{V }({\bar{\text{BCP}}}_{2})\;(1/4)\text{ V }({\bar{\text{P}}}_{2})$$

(20) $$\begin{array}{c} \text{V}l=\text{V}({\bar{\text{P}}}_{1})+V({\bar{P}}_{2})+V({\bar{\text{F}}}_{1})+16V({\bar{\text{F}}}_{2})+16\text{V }({\bar{\text{BCP}}}_{1})\\ +16\text{V }({\bar{\text{BCP}}}_{2}) \end{array}$$

Where, $\text{V }({\bar{\text{P}}}_{1})$ , $\text{V }({\bar{\text{P}}}_{2})$ , ${\text{V }(\text{F}}_{1})$ , ${\text{V }(\text{F}}_{2})$ , $\text{V }({\bar{\text{BCP}}}_{1})$ and $\text{V }({\bar{\text{BCP}}}_{2})$ are the variances of the P_1_, P_2_, F_1_, F_2_, BCP_1_, and BCP_2_ generations, respectively.

The significance of the genetic parameters was tested using the t-test, similar to that used in the scaling test. Initially, the standard error for each component was calculated by finding the square root of the respective variance.

The variance components in the absence of epistasis were computed to determine the genetic parameters using the following formulas suggested by Mather (1949):


(21) $$\text{Heritable fixable variance }\left(D\right)=4{\text{VF}}_{2}–2\;({\text{VBCP}}_{1}+{\text{VBCP}}_{2})$$

(22) $$\text{Heritable non-fixable variance }\left(\text{H}\right)=4{\text{VF}}_{2}–1/2\text{VD}–\text{VE}$$

(23) $$\begin{array}{c} \text{Non-heritable non-fixable variance }\left(\text{E}\right)\\ =1/3\;({\text{VP}}_{1}+{\text{VP}}_{2}+{\text{VF}}_{1}) \end{array}$$

Where F_1_ is the first filial generation, F_2_ is the second filial generation, BCP_1_ is the backcross population derived from parent 1, and BCP_2_ is the backcross population derived from parent 2.

The number of genes responsible for MLB disease resistance and maturity traits were calculated using the following formula (Sekhar et al., 2015):


(24) $$\text{n}={\left({\bar{\text{P}}}_{1}-{\bar{\text{P}}}_{2}\right)}^{2}/8({\text{VF}}_{2}-{\text{VF}}_{1})$$

Where n is number of genes; $\left({\bar{\text{P}}}_{1}\right)$ and $\left({\bar{\text{P}}}_{2}\right)$ are parent means; and ${\text{V }(\text{F}}_{1})$ and ${\text{V }(\text{F}}_{2})$ are the variances of first filial generation and second filial generation, respectively.

### 2.4 Heritability, degree of dominance, and correlation coefficient

Both heritability types, i.e., broad sense (h^2bs^) and narrow sense (h^2ns^), were estimated according to Warner (1952). The degree of dominance, calculated as the square root of the ratio between dominance variance (H) and additive variance (D), was determined following the method of Robinson et al. (1949). Furthermore, Pearson’s correlation coefficient was estimated between PDI and maturity-related traits.


(25) $$\begin{array}{c} {\text{h}}_{bs}^{2}=\frac{\text{VF}2-\frac{\text{VP}1+\text{VP}2+\text{VF}1}{3}}{\text{VF}2}\times 100\\ {\text{h}}_{bs}^{2}=\frac{2\times \text{VF}2-\left(\text{VBCP}1+\text{VBCP}2\right)}{\text{VF}2}\times 100 \end{array}$$

(26) $$\text{Degree of dominance}=\sqrt{\text{H}/\text{D}}$$

Where VP_1_, VP_2_, VF_1_, VBCP_1_, VBCP_2_, and VF2 are the variances of P_1_, P_2,_ F_1_, BCP_1_, BCP_2_, and F_2_ respectively.

## 3 Results

### 3.1 Variability for targeted traits

**Figure 1:**
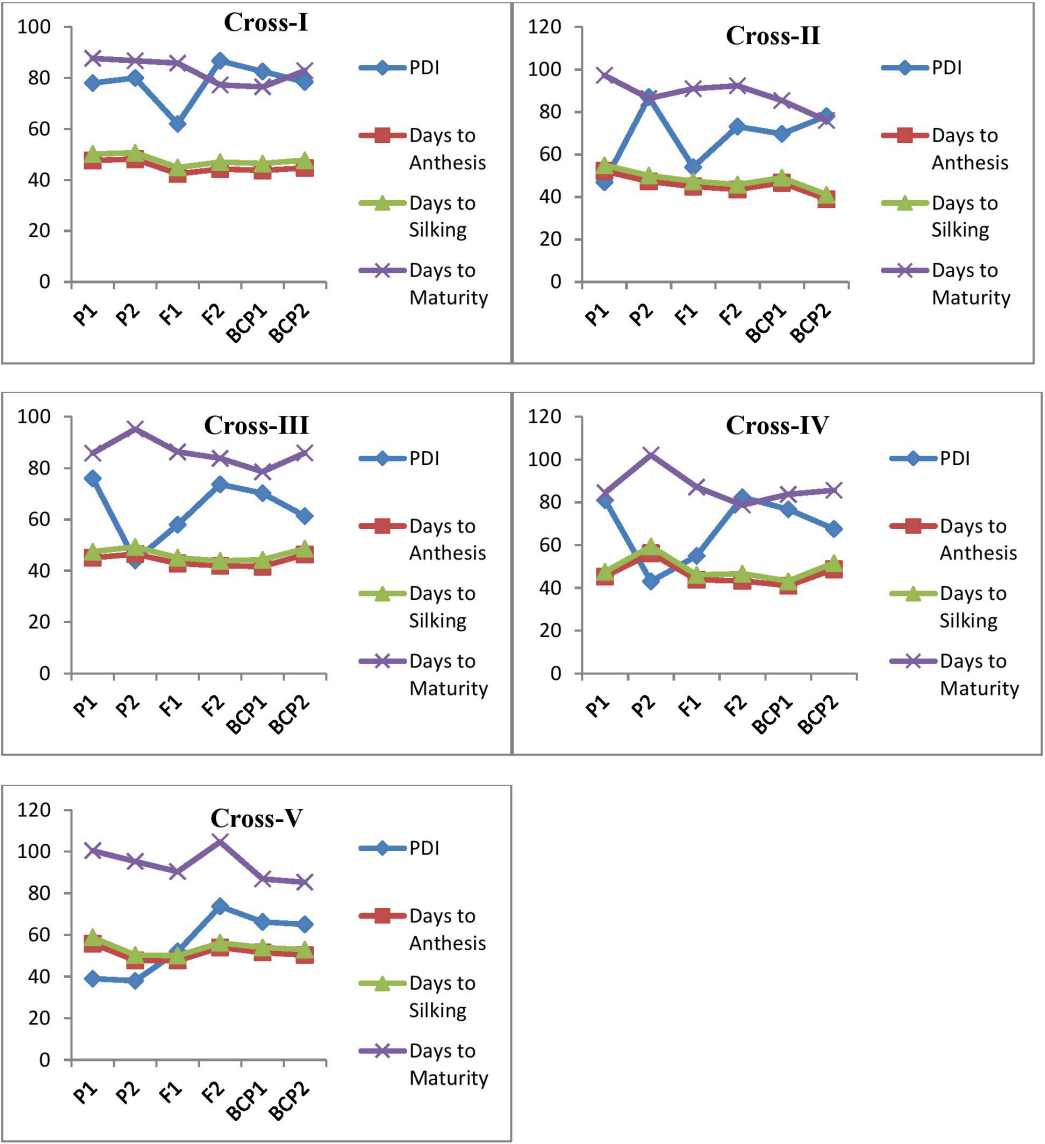
Mean performance of parents (P1 and P2), F1, F2 and backcrosses (BCP1 and BCP2) for PDI values of MLB reaction, days to anthesis, silking, and maturity. The cross I represents HKI 4C4B (S) ×ESM113 (S), Cross II- CML269-1 (R) × HKI4C4B (S), Cross III- HKI4C4B (S) × CML269 (R), Cross IV- ESM113 (S) × P72c1Xbrasil1177-2 (R) and Cross V-P72c1Xbrasil1177-2 (R) × CML269 (R).

The mean and variance data for the parents, F_1_, F_2_, and backcross generations (BC_1_P_1_ and BC_1_P_2_) across five crosses are summarized in **Table 1** and depicted in **Figure 1**. In MLB, the mean PDI of the parents ranged from 38% (P72c1Xbrasil1177-2) to 87% (HKI4C4B). For F_1_, the mean PDI ranged from 52% to 64%. For the backcross populations, the mean PDI values ranged from 64.76% to 83.51%. As for the F_2_ populations, the mean PDIs ranged from 73.19% to 86.72%. For other traits, such as days to 50% anthesis, silking, and maturity, the mean values for the parents ranged from 45.40 (HKI4C4B, early flowering) to 56.20 (P72c1Xbrasil1177-2, late flowering), 47.10 (HKI4C4B) to 59.40 (P72c1Xbrasil1177-2), and 84.50 (ESM113) to 102.80 (P72c1Xbrasil1177-2), respectively. Days to 50% anthesis, silking, and maturity (recorded as the number of days) varied from 42.8 to 48.70, 44.4 to 50.0, and 86.30 to 91.0 for the F_1_ populations, respectively (**Table 1**). In the F_2_ populations, they ranged from 41.83 to 54.00 for days to 50% anthesis, 43.92 to 56.15 for silking, and 77.37 to 104.66 for maturity. In the backcross populations, the mean values for days to anthesis, silking, and maturity ranged from 39.16 to 50.80, 41.34 to 53.25, and 76.52 to 87.25, respectively (**Table 1**). The F_1_ of the cross between S and S exhibited moderate susceptibility; however, the F_2_ (86.72%) and backcross populations (83.51%) displayed higher susceptibility. CML 269-1 and P72c1Xbrasil1177-2 showed moderate resistance to MLB with medium to late flowering. HKI4C4B and ESM 113, however, showed highly susceptible reactions to MLB disease with early to medium flowering durations. An almost moderate resistance response was observed in the F_1_ of the cross between R and R. The R and S reciprocal crosses did not produce significant differences in MLB disease response in F_1_s. The backcross and F_2_ populations of resistant and susceptible crosses showed relatively susceptible reactions for MLB disease. Furthermore, the flowering and maturity traits showed clear-cut heterosis towards earliness, ranging from 3–7 days for anthesis and silking and up to 15 days for maturity (**Table 1**). Variance analysis revealed that the F_2_ generation exhibited significantly higher variability compared to the F_1_, BCP_1_, and BCP_2_ generations, indicating substantial genetic variation and segregation across all the traits evaluated.

**Table 1:** Estimates of mean for PDI, days to anthesis, days to silking, and days to maturity.

Cross	Generation	PDI		Days to anthesis		Days to silking		Days to maturity	
		Mean	VAR	Mean	VAR	Mean	VAR	Mean	VAR
Cross I	P1	77.80	14.18	47.20	4.62	51.20	2.40	87.20	2.62
	P2	80.80	6.84	47.00	5.78	51.80	4.62	86.50	5.83
	F1	64.00	4.44	42.80	5.07	44.40	4.71	87.50	3.61
	F2	86.72	34.37	44.24	11.60	46.99	15.15	77.37	13.28
	BCP1	83.51	15.73	43.70	8.94	46.25	11.71	76.52	8.23
	BCP2	77.29	36.61	43.89	9.05	46.45	9.44	80.32	16.91
Cross II	P1	48.00	6.67	51.80	3.29	54.20	3.73	96.70	6.68
	P2	87.50	6.94	47.10	2.77	49.70	3.57	86.90	6.77
	F1	53.00	62.22	44.90	11.43	47.50	13.83	91.00	15.11
	F2	73.70	77.20	44.05	16.54	46.48	18.87	92.29	32.62
	BCP1	69.41	49.65	47.36	21.90	49.95	26.52	86.69	27.11
	BCP2	73.62	56.26	39.16	3.62	41.34	4.53	77.47	19.19
Cross III	P1	70.00	44.44	45.40	8.49	47.10	7.21	86.00	25.11
	P2	50.00	67.78	45.60	4.49	48.30	3.12	90.40	10.93
	F1	54.00	48.89	43.50	7.61	45.50	9.83	86.30	10.23
	F2	73.71	113.76	41.83	17.00	43.92	21.39	83.94	38.26
	BCP1	67.41	97.35	42.66	13.87	45.46	13.89	81.75	43.47
	BCP2	64.76	78.32	46.20	14.31	48.59	15.84	87.25	17.39
Cross IV	P1	83.00	6.67	45.30	4.01	47.50	4.28	84.50	14.72
	P2	42.40	5.60	56.20	3.96	59.40	3.38	102.10	23.43
	F1	52.50	6.94	43.80	3.07	46.10	2.54	87.10	8.32
	F2	82.41	79.00	43.27	20.80	46.64	34.84	78.74	39.92
	BCP1	76.48	41.92	41.05	17.68	43.16	18.07	83.72	54.05
	BCP2	67.42	77.25	48.76	19.32	51.69	24.88	85.60	5.07
Cross V	P1	39.30	2.68	55.90	1.43	58.50	2.72	102.80	4.62
	P2	38.00	6.67	47.40	3.38	51.20	1.51	94.50	0.50
	F1	54.00	21.11	48.70	1.34	50.00	4.67	88.30	18.01
	F2	73.19	105.39	53.99	10.04	56.15	8.93	104.66	33.81
	BCP1	66.34	53.91	50.80	5.54	53.25	4.84	86.63	15.96
	BCP2	67.99	68.08	49.96	8.88	52.57	9.89	85.15	28.57

Cross I, HKI 4C4B (S) × ESM113 (S); Cross II, CML269-1 (R) × HKI4C4B (S); Cross III, HKI4C4B (S) × CML269 (R); Cross IV; ESM113 (S) × P72c1Xbrasil1177-2 (R); and Cross V; P72c1Xbrasil1177-2 (R) × CML269 (R).

P_1_, parent 1; P_2_, parent 2; F_1_, first filial generation; F_2_, second filial generation; BCP_1_, backcross population derived from parent 1; BCP_2_, backcross population derived from parent 2; PDI, percent disease index; VAR, variance.

### 3.2 Scaling test

The scaling tests (A, B, C, and D) for the four traits indicated significant deviations from the additive-dominance model (**Table 2**), suggesting the presence of epistasis (non-allelic interactions) except for non-significance of scales B and C for days to anthesis in Cross I; non-significance of scale A for days to anthesis and silking and scale C for days to maturity in Cross II; and non-significance of scale A for days to silking, and of scales B and D for days to maturity in Cross III. For each characteristic, at least one of the four scaling tests (A, B, C, and D) revealed significance, indicating that the additive-dominance model is insufficient to explain the epistasis and that epistasis or non-allelic interactions regulate the expression of all the targeted traits (**Table 2**).

**Table 2:** Estimation of the scaling value of A, B, C, and D for PDI, days to anthesis, days to silking, and days to maturity in different crosses.

Cross	PDA				Days to anthesis				Days to silking				Days to maturity			
	A	B	C	D	A	B	C	D	A	B	C	D	A	B	C	D
Cross I	25.21**	9.77**	60.28**	12.64**	-2.69**	-1.90	-2.62	0.98*	-3.09**	-3.30**	-3.82*	1.28**	-21.66*	-13.35**	-39.22**	-2.10**
Cross II	37.81**	6.74*	51.25**	3.34*	-1.97	-13.68**	-12.50**	1.58*	-1.80	-14.51**	-12.97**	1.67*	-14.31**	-22.96**	3.57	20.42**
Cross III	10.81**	22.51**	63.82**	15.24**	-3.78*	3.50**	-10.69**	-5.20**	-1.68	3.38*	-10.72**	-6.20**	-8.80**	-2.20	-13.24**	-1.11
Cross IV	17.45**	39.94**	99.24**	20.92**	-7.00**	-2.48**	-16.03**	-3.27**	-7.28**	-2.12*	-12.52**	-1.56*	-4.16*	-18.00**	-45.84**	-11.83**
Cross V	39.38**	43.97**	109.48**	13.05**	-3.00**	3.81**	15.27**	7.23**	-2.00*	3.93**	14.90**	6.49**	-17.83**	-12.49**	44.73**	37.52**

Cross I, HKI 4C4B (S) × ESM113 (S); Cross II, CML269-1 (R) × HKI4C4B (S); Cross III, HKI4C4B (S) × CML269 (R); Cross IV; ESM113 (S) × P72c1Xbrasil1177-2 (R); and Cross V; P72c1Xbrasil1177-2 (R) × CML269 (R).

*Significant at P<0.05, **Significant at P<0.01. PDI, Percent Disease Index.

### 3.3 Genetic effects

#### 3.3.1 Maydis leaf blight disease

The mean values of six generations for all four traits were partitioned into six types of genetic effects, all of which were significant (*P*< 0.05**).** These effects were estimated using the six-parameter model proposed by Jinks and Jones (1958). The mean effect was consistently higher than the other genetic effects evaluated, such as additive effect (d), dominance effect (h), additive × additive interaction effect (i), additive × dominance interaction effect (j), and dominance × dominance interaction effect (l), across the five crosses (**Table 3**).

**Table 3:** Estimation of the direct and interaction gene effects for PDI, days to anthesis, days to silking, and days to maturity in different crosses.

Cross	Six-parameter model											
	PDA						Days to silking					
	m	d	h	i	j	l	m	d	h	i	j	l
Cross I	86.72**	6.21**	-40.58**	-25.28**	7.71**	-9.70**	46.99**	-0.19	-9.67**	-2.5	0.11	8.97**
Cross II	73.18**	-4.21**	-21.44**	-6.69*	15.53**	-37.86**	46.48**	8.60**	-7.79**	-3.34*	6.35**	19.67**
Cross III	73.70**	2.64*	-37.99**	-30.49**	-5.85*	-2.82	43.92**	-3.13**	10.21**	12.41**	-2.53**	-14.11**
Cross IV	82.41**	9.05**	-52.04**	-41.84**	-11.24**	-15.55**	46.64**	-8.52**	-4.22*	3.12*	-2.57**	6.28*
Cross V	73.69**	-1.64	-10.76**	-26.11**	-2.29*	-57.24**	56.15**	0.68**	-17.83**	-12.98**	-2.96**	11.05**
	Days to anthesis						Days to maturity					
Cross I	44.24**	-0.29	-6.37**	-1.97*	-0.39	6.56**	77.36**	-3.80**	4.85**	4.20**	-4.15**	30.80**
Cross II	44.05**	8.20**	-7.71**	-3.16*	5.85**	18.82**	92.29**	9.22**	-41.65	-40.85**	4.32**	78.13**
Cross III	41.82**	-3.54*	8.41**	10.41**	-3.64*	-10.14	83.93**	-5.50**	0.33	2.23	-3.30*	8.77*
Cross IV	43.26**	-7.70**	-0.40	6.54**	-2.25**	2.94	78.73**	-1.87**	17.46**	23.66**	6.92**	-1.48
Cross V	53.99**	0.84*	-17.41**	-14.46**	-3.40**	13.65**	104.65**	1.48*	-85.40**	-75.05**	-2.66**	105.38**

Cross I, HKI 4C4B (S) × ESM113 (S); Cross II, CML269-1 (R) × HKI4C4B (S); Cross III, HKI4C4B (S) × CML269 (R); Cross IV; ESM113 (S) × P72c1Xbrasil1177-2 (R); and Cross V; P72c1Xbrasil1177-2 (R) × CML269 (R).

*Significant at P<0.05, **Significant at P<0.01. m, mean effect; d, additive effect; h, dominance effect; i, additive × additive effect; j, additive × dominance effect; l, dominance × dominance effect, PDI, percent disease index.

Among the cross-wise direct and interallelic genetic effects on the PDI, all six genetic effects were significant (*P*< 0.01) in Cross I (**Table 3**). The dominance genetic effect was 85% greater than the additive genetic effect, while among interaction effects, the additive × additive effect exceeded the dominance × dominance effect by 62%. In Cross II, both the additive and dominance genetic effects were significant (*P*< 0.01**),** with the dominance effect being 80% higher than the additive effect. Among the non-allelic interactions, the additive × dominance and dominance × dominance effects were significant (*P*< 0.01**),** as was the additive × additive effect (*P*< 0.05**).** The dominance × dominance effect exceeded the additive × additive effect by 82%. In Cross III, both the additive (*P< 0.05*) and dominance genetic effects were significant, wherein the magnitude of the dominance genetic effect was 93% higher than the additive genetic effect. Among the interaction effects, both the additive × additive (*P*< 0.01) and additive × dominance (*P*< 0.05) genetic effects were significant, while dominance × dominance was non-significant. The additive × additive effect was 81% higher than the additive × dominance genetic effect. In Cross IV, all six genetic effects were significant (*P*< 0.01**).** The dominance genetic effect was 83% higher than the additive effect, while the additive × additive interaction effect exceeded the dominance × dominance interaction effect by 63%. In Cross V, except for the additive genetic effect, all six types of genetic effect were significant (*P< 0.05*). The dominance × dominance genetic effect was 54% higher than the additive × additive genetic effect.

#### 3.3.2 Days to anthesis

Days to anthesis showed a highly significant (*P<* 0.01) dominant effect over all the other genetic effects studied. In the case of Cross I for days to anthesis, the dominance genetic effect and dominance × dominance interaction effects were highly significant (*P<0.01*) although the additive × additive effect was significant at *P< 0.05*. The dominance × dominance effect exceeded the additive × dominance effect by 94%. The opposite directions of the dominance genetic effect and dominance × dominance interaction effects suggested the presence of duplicate epistasis. In Cross II, all direct genetic and interaction effects were observed to be significant at *P< 0.01* and *P< 0.05* levels, wherein the dominance × dominance effect was 69% higher than the additive × dominance effect. The contrasting signs of the dominant genetic and dominant × dominant interaction effects suggested the presence of duplicate epistasis. In Cross III, all the direct genetic and gene interaction effects were significant (*P< 0.01* and *P< 0.05*) except for the dominance × dominance interaction effects. The dominance genetic effect was 52% higher than the additive genetic effects. In Cross IV, among the direct genetic effects, only the additive genetic effect was significant (*P*< 0.01**).** For the interaction effects, both the additive × additive and additive × dominance effects were highly significant (*P*< 0.01**),** with the additive × additive effect being 66% greater than the additive × dominance effect. In Cross V, all the direct genetic and interaction effects were significant (*P< 0.01* and *P< 0.05*). The dominance × dominance effect was 86% higher than the additive × dominance effect. The contrasting sign of the dominant genetic effect and dominance × dominance interaction effects suggest the presence of duplicate epistasis.

#### 3.3.3 Days to silking

The mean effect of days to silking was highly significant (*P< 0.01*) and higher than all the other genetic effects evaluated, including the interaction effects. In Cross I, among the direct genetic effects, only the dominance genetic effect was significant (*P*< 0.01). For the interaction effects, the dominance × dominance interaction was significant (*P*< 0.01) and was 99% higher than the additive × dominance interaction. The opposite signs of the dominance genetic effect and the dominance × dominance interaction indicated duplicate gene action. In Cross II, all the direct genetic and interaction effects were significant (*P*< 0.01 and *P*< 0.05). The additive genetic effect was 10% higher than the dominance genetic effect. Among the interaction effects, the dominance × dominance interaction was 68% greater than the additive × dominance interaction. The opposite signs of the dominance genetic effect and dominance × dominance interaction again indicated duplicate gene action. In Cross III, all the direct genetic and interaction effects were significant (*P*< 0.01). The dominance genetic effect exceeded the additive genetic effect by 70%. Among the interaction effects, the dominance × dominance interaction was 83% higher than the additive × dominance interaction. The contrasting values of the dominance genetic effect and dominance × dominance interaction suggested duplicate epistasis. In Cross IV, all direct genetic and interaction effects were significant (*P*< 0.01 and *P*< 0.05). The dominance genetic effect was 90% greater than the additive genetic effect, while the dominance × dominance interaction was 59% higher than the additive × dominance interaction. The opposing directions of the dominance genetic effect and dominance × dominance interaction again suggested duplicate epistasis. In Cross V, all direct the genetic and interaction effects were significant (*P*< 0.01). The dominance genetic effect was 96% higher than the additive genetic effect, and the dominance × dominance interaction was 74% higher than the additive × dominance interaction. The contrasting directions of the dominance genetic effect and dominance × dominance interaction indicated duplicate epistasis.

#### 3.3.4 Days to maturity

The mean effect of days to maturity was highly significant (*P<* 0.01) and the dominance effect was higher than all other genetic effects, including the interactions. In Cross I, all the direct genetic effects and interactions were significant (*P*< 0.01). The dominance genetic effect was 22% higher than the additive genetic effect, while the dominance × dominance interaction was 87% greater than the additive × dominance interaction. All the interaction effects were significant (*P*< 0.01) in Cross II, but among the direct effects, only the additive genetic effect was significant (*P*< 0.01). The dominance × dominance interaction was 94% higher than the additive × dominance interaction. The contrasting directions of the dominance genetic effect and dominance × dominance interaction suggested duplicate epistasis. In Cross III, among the direct genetic effects, only the additive genetic effect was significant (*P*< 0.01). The dominance × dominance interaction was 62% greater than the additive × dominance interaction. In Cross IV, both the additive and dominance genetic effects were significant (*P*< 0.01) among the direct effects. The interaction effects, including additive × additive and additive × dominance, were also significant (*P*< 0.01). In Cross V, both the additive (*P*< 0.05) and dominance (*P*< 0.01) genetic effects were significant among the direct effects. All the interaction effects were significant (*P*< 0.01). The dominance × dominance interaction effect exceeded the additive × dominance interaction effect by 97%.

### 3.4 Components of genetic variance, heritability, correlation, and number of genes

The generation variance components, as described by Mather (1949), are presented in **Table 4**. The analysis revealed that in Cross I, the dominance variance was predominant compared to the additive and environmental variances for PDI and days to maturity, whereas the additive variance was predominant over the dominance and environmental variances for days to anthesis and days to silking. In Cross II, the dominance variance was more pronounced for days to silking, while the additive variance dominated the dominance and environmental variances for PDI, days to anthesis, and days to maturity. In Cross III, the dominance variance was predominant for days to anthesis, while additive variance surpassed the dominance and environmental variances for PDI, days to silking, and days to maturity. In Cross IV, the dominance variance was higher for PDI and days to anthesis, whereas the additive variance was more significant for days to silking and days to maturity. In Cross V, the dominance variance was predominant for days to silking, while the additive variance was more significant for PDI, days to anthesis, and days to maturity. The H/D ratio for all the traits (**Table 4**), ranged from 0.67 to 3.32 in Cross I, 0.37 to 1.23 in Cross II, 0.49 to 1.21 in Cross III, 0.59 to 2.33 in Cross IV, and 0.38 to 1.34 in Cross V, indicated the dominance for each cross.

**Table 4:** Estimates of generation variance components and number of effective genes and heritability for PDI, days to anthesis, days to silking, and days to maturity in different crosses.

Generation variance components	PDI	Days to anthesis	Days to silking	Days to maturity
Cross I				
E	8.48	4.72	3.91	4.02
D	32.79	10.39	18.30	2.84
H	37.92	6.68	8.34	31.34
No. of genes	0.226	0.025	0.069	0.133
H/D	1.07	0.80	0.67	3.32
h^2bs^%	75.39	55.60	74.17	69.70
h^2ns^%	47.74	44.62	60.36	10.62
Cross II				
E	25.27	5.82	7.04	9.51
D	96.96	15.10	13.37	37.89
H	13.74	12.62	20.54	16.63
No. of genes	0.005	0.007	0.006	5.780
H/D	0.37	0.91	1.23	0.66
h^2bs^%	67.25	64.77	62.77	70.83
h^2ns^%	62.80	45.67	35.41	58.09
Cross III				
E	53.70	6.86	6.72	15.42
D	103.73	11.64	26.11	31.29
H	32.77	17.25	6.46	28.73
No. of genes	1.048	0.213	0.180	0.897
H/D	0.56	1.21	0.49	0.95
h^2bs^%	52.79	59.64	68.58	59.67
h^2ns^%	45.58	34.25	61.00	40.88
Cross IV				
E	6.40	3.67	3.40	15.49
D	77.67	9.20	53.43	41.44
H	135.04	50.08	18.88	14.83
No. of genes	0.002	2.536	0.003	0.300
H/D	1.31	2.33	0.59	0.59
h^2bs^%	91.89	82.33	90.25	61.19
h^2ns^%	49.16	22.11	76.68	51.90
Cross V				
E	10.15	2.05	2.96	7.71
D	177.60	11.32	6.24	46.18
H	25.75	9.29	11.35	12.02
No. of genes	0.023	0.054	0.042	0.034
H/D	0.38	0.90	1.34	0.51
h^2bs^%	90.36	79.59	66.77	77.19
h^2ns^%	84.25	56.33	34.86	68.32

Cross I, HKI 4C4B (S) × ESM113 (S); Cross II, CML269-1 (R) × HKI4C4B (S); Cross III, HKI4C4B (S) × CML269 (R); Cross IV; ESM113 (S) × P72c1Xbrasil1177-2 (R); and Cross V; P72c1Xbrasil1177-2 (R) × CML269 (R).

E, Environmental variance; D, additive variance; H, dominance variance; PDI, percent disease index; h^2bs^%, broad-sense heritability; h^2ns^%, narrow-sense heritability.

The estimated minimum number of genes (**Table 4**) contributing to PDI, days to anthesis, days to silking, and days to maturity in Cross I were 0.226, 0.025, 0.069, and 0.133, respectively, while in Cross II, these values were 0.005, 0.007, 0.006, and 5.780, respectively. Similarly, the minimum number of genes estimated for these traits in Cross III was 1.048, 0.213, 0.180, and 0.897, respectively; 0.002, 2.536, 0.003, and 0.300, respectively, in Cross IV; and 0.023, 0.054, 0.042, and 0.034, respectively, in Cross V.

The broad-sense heritability (**Table 4**) ranged from 52.79% to 91.89% for PDI, 55.60% to 82.33% for days to anthesis, 62.77% to 90.25% for days to silking, and 59.67% to 77.19% for days to maturity, whereas the narrow-sense heritability ranged from 45.58% to 84.25% for PDI, 22.11% to 56.33% for days to anthesis, 34.86% to 76.68% for days to silking, and 10.62% to 68.32% for days to maturity, indicating the differential contribution of additive genetic variance to the traits.

Pearson’s correlation analysis, conducted using the mean values of F_2_, BCP_1_, and BCP_2_ generations across all five crosses (**Table 5**), showed that PDI was significantly negatively associated with days to anthesis (−0.20^∗∗^), days to silking (−0.18^∗∗^), and days to maturity (−0.27^∗∗^). Days to maturity showed a significant positive correlation with days to silking (0.43^∗∗^) and days to anthesis (0.47^∗∗^), while days to anthesis exhibited a strong positive correlation with days to silking (0.93∗∗).

**Table 5:** Correlations among PDI, days to anthesis, days to silking, and days to maturity.

	PDI	DA	DS	DM
PDI	1.000	−0.20∗∗	−0.18∗∗	−0.27∗∗
DA		1.000	0.93∗∗	0.47∗∗
DS			1.000	0.43∗∗
DM				1.000

**Significant at P<0.01. PDI, percent disease index; DA, days to anthesis; DS, days to silking; DM, days to maturity.

## 4 Discussion

The most sustainable and cost-effective way to manage crop diseases is to develop disease-resistant cultivars, which requires a thorough understanding of disease genetics. Additionally, key maturity traits, such as days to anthesis, silking, and maturity, play a crucial role in ensuring better maize productivity in stress-prone ecologies. Resistance to MLB has been established as a quantitative trait (Burnett and White, 1985), emphasizing the importance of studying gene effects to facilitate the development of disease-resistant maize cultivars. Studies on MLB resistance using generation mean analysis have been reported by Jeevan et al. (2020) and Vasmatkar et al. (2022). The biometrical approach provides critical genetic insights for designing breeding strategies that leverage gene interactions and effects across successive breeding generations (Jeevan et al., 2020; Vasmatkar et al., 2022). In this study, the F_1_ generation exhibited moderate resistance to MLB across all crosses, except for Cross I (S × S), where it was found to be susceptible. Notably, the average PDI values of the F_1_ generation were generally lower than the mid-parent values, except in Cross V, where the mid-parent PDI value matched the parental resistance levels. The F_1_ generation showed no significant differences compared to the backcross generations (BCP_1_ and BCP_2_), favoring a dominance gene action for MLB resistance. The dominant nature of resistance suggests the potential for heterosis breeding in MLB resistance on a commercial scale. This aligns with findings by Jeevan et al. (2020). In the present study, the non-segregating parental (P_1_ and P_2_) and F_1_ generations exhibited lower phenotypic variation due to their homogenous genetic nature and limited micro and macro environmental effects. The large variation in segregating generations (F_2_ and backcrosses) can be attributed to the segregation of loci and modifiers associated with MLB resistance and susceptibility. Similar patterns of phenotypic variation across different generations have been reported in previous studies (Sullenberger et al., 2018), which further corroborates the findings. There was also a much stronger dominance effect in the flowering and maturity traits, suggesting that hybrid technology can be used effectively for their breeding. The negative dominance effect observed in this study is indicative of heterosis towards earliness. These results align with earlier findings that reported significant dominance effects for days to silking and days to maturity (Al-Baidhani et al., 2022). Similarly, Ali et al. (2018) and Sher et al. (2012) also identified higher and significant dominance effects for days to anthesis and silking in maize. These results highlight the importance of understanding the genetics and variability in targeted traits for effective breeding strategies.

The scaling test revealed significant values across all crosses and traits, indicating the presence of epistatic interactions in the studied traits (Pujar et al., 2022). Epistasis refers to non-allelic interactions between genes (Phillips, 1998; Boubacar Gaoh et al., 2020). This can help in interpreting the role of breeding systems in crop evolution and developing suitable cultivars (Hallauer and Miranda, 1981). In this study, a significant additive × additive interaction effect was observed for MLB disease resistance. As a result of additive × additive interactions, the early generation selection becomes more favorable while breeding for traits of interest, which are more heritable. Similar findings have been reported in previous studies on MLB resistance in maize (Jeevan et al., 2020). In the case of days to anthesis, silking, and maturity, the dominance × dominance interaction consistently showed significant effects in the majority of the crosses. Dominance × dominance interactions have been reported in previous studies for flowering and maturity traits in maize (Shankar et al., 2022). In breeding, this kind of interaction favors selection during lateral generations to acquire more genetic gain. It is also more appropriate to breed for hybrids, synthetics, and population improvement in the case of a dominance × dominance interaction. A comprehensive understanding of gene action and interactions is crucial for selecting breeding methods that effectively harness genetic variance.

A low number of genes governing MLB resistance and maturity-related traits were detected in this study. This is possibly because the traits are polygenic, and epistatic interactions appear to be more complex. When using generation mean analysis, it is generally difficult to detect genes with minor effects. Thus, we suggest using advanced approaches such as QTL mapping, genome-wide association mapping, and mixed model analysis to detect the effective numbers of genes. This observation aligns with a previous study on MLB resistance (Jeevan et al., 2020) and resistance to *Sesamia inferens* infestation in maize (Sekhar et al., 2015).

Moderate to high narrow- and broad-sense heritability were observed across all crosses, indicating the potential for effective selection to improve these targeted traits. Similar findings have been reported in previous studies on maize by Ali et al. (2018); Khalil et al. (2010), and Santosh et al. (2012). A significant negative correlation was observed between MLB resistance and maturity-related traits. However, the correlations among the maturity-related traits (days to anthesis, silking, and maturity) were significantly positive. These findings suggest that, in contrast to shorter-duration genotypes, longer-duration genotypes appear to be more resistant to MLB disease. This result aligns with previous studies in maize by Chandrashekara et al. (2014); Ali et al. (2014), and Kumar et al. (2016). Selection for one flowering or maturity trait can also lead to improvements in another, as they are positively correlated with each other (Kumar et al., 2016). All these findings and relationships can provide valuable insights for selecting desirable genotypes and optimizing breeding strategies. Furthermore, the genetic interrelationship between disease susceptibility and maturity-related traits provides valuable insights for breeding programs aiming to improve these traits.

## 5 Conclusion

In maize, MLB resistance and maturity-related traits exhibit quantitative inheritance. MLB resistance is significantly influenced by non-fixable heritable direct effects and fixable heritable (additive × additive) interactions, whereas flowering/maturity traits exhibit a higher magnitude of significant non-fixable (dominance) effects. Hence, transgressive segregants can be obtained with varying levels of MLB resistance and maturity. Recurrent selection and hybrid breeding strategies could be explored to maintain the favorable heterozygous allele combinations in the populations. In our study, a low number of genes were detected through GMA, indicating the need to use QTL mapping and genome-wide association studies to detect a significantly greater number of genomic regions that regulate MLB resistance and flowering/maturity traits. The establishment of a negative correlation between MLB resistance and flowering/maturity traits signifies the need to develop long-duration hybrids to effectively manage the MLB disease in maize, minimizing yield losses to farmers. In summary, this study provides the genetic basis of MLB resistance in maize and establishes that long-duration hybrids are resistant to MLB, guiding researchers to attain higher genetic gains for MLB resistance and higher yields in maize.

## Data Availability

The original contributions presented in the study are included in the article/**Supplementary Material**. Further inquiries can be directed to the corresponding author.
