# Supplementary figures and images for: Understanding the genetic basis of resistance to maydis leaf blight and maturity-related traits in corn
Source: Front Plant Sci. 2025 Mar 26;16:1551940. doi: 10.3389/fpls.2025.1551940 (PMC11979256; doi:10.3389/fpls.2025.1551940)

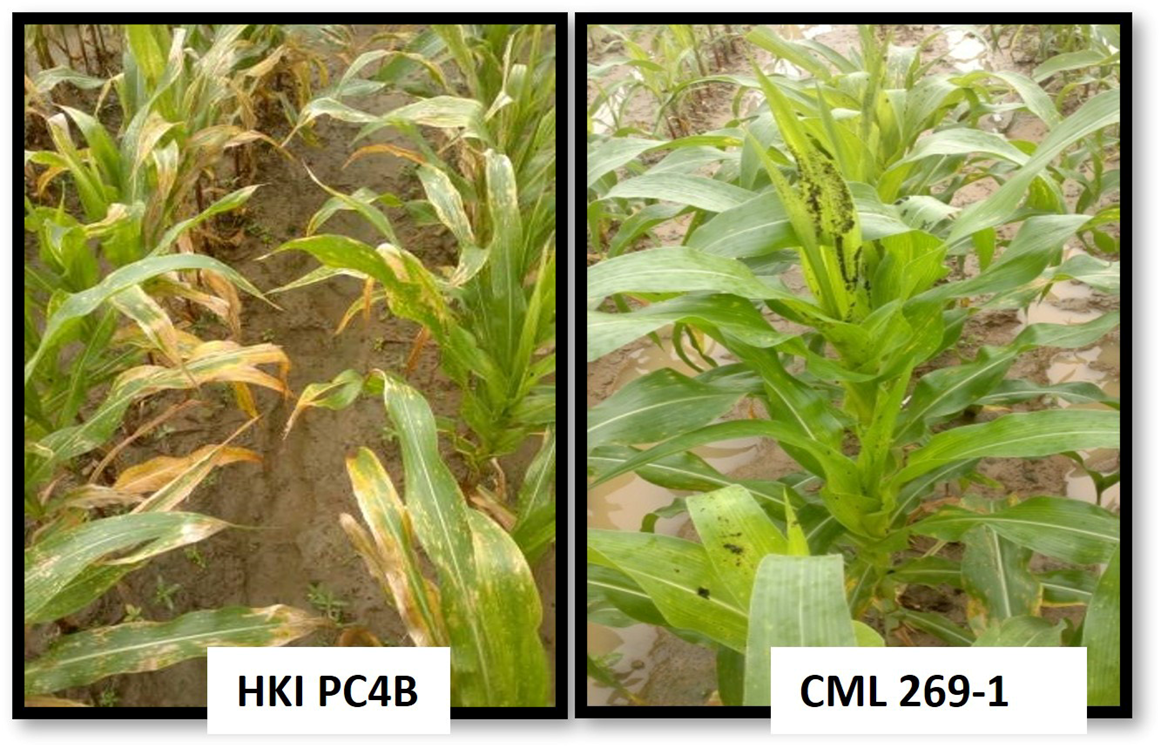


Supplementary Figure 1. Pictorial view of MLB symptoms as HKI PC4B (Susceptible) and CML 269-1 (Resistant)

Supplement: Supplementary file 1 [file SupplementaryFile1.docx]
