# Supplementary material for: Understanding the genetic basis of resistance to maydis leaf blight and maturity-related traits in corn
Source: Front Plant Sci. 2025 Mar 26;16:1551940. doi: 10.3389/fpls.2025.1551940 (PMC11979256; doi:10.3389/fpls.2025.1551940)
Supplement: Supplementary file 2 [file Table1.docx]

**Supplementary Table 1** Parentage of Maize inbred lines (P_1_ and P_2_) involved in the study of generation mean analysis

| **Cross** | **P_1_** | **P_2_** | **No. of Plant evaluated per generation** | | | | | |
| --- | --- | --- | --- | --- | --- | --- | --- | --- |
|  |  |  | P_1_ | P_2_ | F_1_ | F_2_ | BC_1_P_1_ | BC_1_P_1_ |
| I | HKI 4C4B (S) | ESM113 (S) | 10 | 10 | 10 | 311 | 154 | 238 |
| II | CML269-1 (R) | HKI4C4B (S) | 10 | 10 | 10 | 160 | 169 | 158 |
| III | HKI4C4B (S) | CML269 (R) | 10 | 10 | 10 | 375 | 79 | 290 |
| IV | ESM113 (S) | P72c1Xbrasil1177-2 (R) | 10 | 10 | 10 | 311 | 264 | 225 |
| V | P72c1Xbrasil1177-2 (R) | CML269 (R) | 10 | 10 | 10 | 184 | 145 | 159 |

**Supplementary Table 2:** The disease scale employed to identify the disease incidence in various generations (Hood et al., 2018)

| **Rating scale** | **Degree of infection (per cent DLA*)** | **PDI**** | **Disease reaction** |
| --- | --- | --- | --- |
| 1.0 | Nil to very slight infection (<10%). | <11.11 | Resistant (R) |
| 2.0 | Slight infection, a few lesions scattered on two  lower leaves (10.1-20%) | 22.22 | (Score: < 3.0)  (DLA:< 30%) |
| 3.0 | Light infection, moderate number of lesions  scattered on four lower leaves (20.1-30%). | 33.33 | (PDI: < 33.33) |
| 4.0 | Light infection, moderate number of lesions  scattered on lower leaves, a few lesions scattered  on middle leaves below the cob (30.1-40%). | 44.44 | Moderately resistant (MR)  (Score: 3.1–5.0) |
| 5.0 | Moderate infection, abundant number of lesions  scattered on lower leaves, moderate number of  lesions scattered on middle leaves below the cob  (40.1-50%) | 55.55 | (PDI: 33.34-55.55) |
| 6.0 | Heavy infection, abundant number of lesions scattered on lower leaves, moderate infection on middle leaves and a few lesions on two leaves above the cob (50.1-60%). | 66.66 | Moderately susceptible  (Score: 5.1-7.0)  (DLA: 50.1-70%) |
| 7.0 | Heavy infection, abundant number of lesions  scattered on lower and middle leaves and  moderate number of lesions on two to four  leaves above the cob (60.1-70%). | 77.77 | (PDI: 55.56-77.77) |
| 8.0 | Very heavy infection, lesions abundant scattered on lower and middle leaves and spreading up to the flag leaf (70.1-80%). | 88.88 | Susceptible (S)  (Score: >7.0)  (DLA: >70%) |
| 9.0 | Very heavy infection, lesions abundant scattered  on almost all the leaves, plant prematurely dried  and killed (>80%). | 99.99 | (PDI: >77.77) |
